# Genome-Wide Analysis of CCA1-Like Proteins in Soybean and Functional Characterization of GmMYB138a

**DOI:** 10.3390/ijms18102040

**Published:** 2017-09-22

**Authors:** Shaomin Bian, Donghao Jin, Ruihua Li, Xin Xie, Guoli Gao, Weikang Sun, Yuejia Li, Lulu Zhai, Xuyan Li

**Affiliations:** College of Plant Science, Jilin University, Changchun 130062, China; shmbian@jlu.edu.cn (S.B.); jindh16@mails.jlu.edu.cn (D.J.); rhli15@mails.jlu.edu.cn (R.L.); xiexin16@mails.jlu.edu.cn (X.X.); ggl2991@163.com (G.G.); sunwk8214@mails.jlu.edu.cn (W.S.); liyj8214@mails.jlu.edu.cn (Y.L.); zhailulu@jlu.edu.cn (L.Z.)

**Keywords:** CCA1-like proteins, MYB, protein interaction, 14-3-3, soybean

## Abstract

Plant CIRCADIAN CLOCK ASSOCIATED1 (CCA1)-like proteins are a class of single-repeat MYELOBLASTOSIS ONCOGENE (MYB) transcription factors generally featured by a highly conserved motif SHAQK(Y/F)F, which play important roles in multiple biological processes. Soybean is an important grain legume for seed protein and edible vegetable oil. However, essential understandings regarding CCA1-like proteins are very limited in soybean. In this study, 54 CCA1-like proteins were identified by data mining of soybean genome. Phylogenetic analysis indicated that soybean CCA1-like subfamily showed evolutionary conservation and diversification. These *CCA1-like* genes displayed tissue-specific expression patterns, and analysis of genomic organization and evolution revealed 23 duplicated gene pairs. Among them, GmMYB138a was chosen for further investigation. Our protein–protein interaction studies revealed that GmMYB138a, but not its alternatively spliced isoform, interacts with a 14-3-3 protein (GmSGF14l). Although GmMYB138a was predominately localized in nucleus, the resulting complex of GmMYB138a and GmSGF14l was almost evenly distributed in nucleus and cytoplasm, supporting that 14-3-3s interact with their clients to alter their subcellular localization. Additionally, qPCR analysis suggested that *GmMYB138a* and *GmSGF14l* synergistically or antagonistically respond to drought, cold and salt stresses. Our findings will contribute to future research in regard to functions of soybean CCA1-like subfamily, especially regulatory mechanisms of *GmMYB138a* in response to abiotic stresses.

## 1. Introduction

MYB (MYELOBLASTOSIS ONCOGENE) transcription factors are a class of key players in the regulatory networks underlying plant growth and development as well as plant responses to stresses. They are characterized by the presence of MYB domain responsible for DNA binding, predominately at the N-terminus [1]. MYB proteins generally contain 1–4 imperfect amino acid repeats (R0, R1, R2, and R3) of approximately 50–53 amino acids. Each repeat forms three α-helices, and the third α-helix acts as DNA recognition site to interact with the major groove of DNA. During DNA contact, two recognition helices generally bind cooperatively to specific DNA motif in their target promoters [2].

MYB-related family is a heterogeneous collection of R1/R2, R3-MYB type proteins, and usually contains a single MYB repeat [2,3]. It can be divided into five subfamilies: CIRCADIAN CLOCK ASSOCIATED1 (CCA1)-like, CAPRICE-like, Telomeric DNA-binding protein-like, I-box-binding factor-like, and R-R-type [3,4]. Among them, CCA1-like proteins constitute major subfamily of MYB-related proteins, and generally harbor the consensus sequences SHAQK(Y/F)F within the single MYB repeat [3,5]. It has been demonstrated that CCA1-like proteins exert important functions in plant. The best known functions of CCA1-like proteins [such as CCA1, LHY (LATE ELONGATED HYPOCOTYL), MYBH (MYB HYPOCOTYL ELONGATION-RELATED), MYBD (MYB-LIKE PROTEIN D), and RVEs (REVEILLE)] are their involvement of circadian rhythm regulation in plants. As an example, CCA1 and its homolog LHY serve as master regulators to initiate and set the phase of circadian clock by binding directly to the evening element (EE) motif in target gene promoters [6,7,8]. Likewise, RVE8/LCL5 can facilitate the expression of the oscillator gene *TIMING OF CAB EXPRESSION1* (*TOC1*) by binding to its promoter region [9]. In addition to the involvement of circadian rhythm regulation, increasing evidence indicated that CCA1-like proteins might perform diverse functions in various biological processes including the biosynthesis of isoflavonoid and anthocyanin, flowering, leaf senescence, hypocotyl elongation, seed germination, hormone signaling pathway, and stress responses [10,11,12,13,14,15,16]. For example, *LHY* and *CCA1* regulate photoperiodic flowering via affecting expression of *FT* gene in Arabidopsis [12,13]; *MYBD* and *RVE8/LCL5* function as positive regulators to be involved in the regulation of anthocyanin biosynthesis [17,18]; *RVE1/2* can control seed dormancy and germination [19]; *MYBH* participates in the regulation of leaf senescence [20]; *CCA1* regulates ROS (Reactive oxygen species) homeostasis and oxidative stress responses [11]; and *OsMYBS3* (*MYB-RELATED PROTEIN S3*) is essential for cold adaptation in rice [21]. It is also noteworthy that different members of CCA1-like subfamily might perform opposing regulatory functions. For instance, *CCA1* represses *TOC1* expression by promoting histone deacetylation, whereas *RVE8/LCL5* might facilitate *TOC1* expression via pronouncing H3 acetylation at the *TOC1* promoter [9]. Similarly, two CCA1-like transcription factors in Arabidopsis, *MYBS1* and *MYBS2*, play opposite roles in regulating glucose and ABA signaling during seed germination and early seedling development [22]. Evidently, CCA1-like MYB transcription factors play important roles in diverse biological processes. However, the comprehensive understandings of CCA1-like subfamily are limited to few plant species such as Arabidopsis and rice [3]. Therefore, identification and characterization of CCA1-like proteins in diverse plant species becomes an important step toward understanding their functional roles.

The regulation of transcription factors at protein level is a vital method for controlling their activity efficiently and precisely. To date, however, our understandings regarding the regulation of CCA1-like MYBs at protein level were remarkably limited. Emerging evidence indicated that protein–protein interactions can affect the regulatory activity of MYB transcription factors [23,24,25]. Thus, digging out the interactors of CCA1-like proteins is of great significance to understand the mechanisms on a rapid on-and-off switch of their activities. 14-3-3s are a group of regulatory proteins that ubiquitously exist in eukaryotes. 14-3-3 Protein was first isolated from brain tissues and numerically designated in terms of column fractionation and electrophoresis mobility. They can modulate the stability, activity, intracellular localization or interaction capability of their client proteins via protein–protein interactions [26,27]. Plant 14-3-3s have been shown to play crucial roles in a wide variety of biological processes including plant development, protein trafficking, ion channel activity, primary metabolism, signal transduction and stress responses [27,28]. Generally, plant 14-3-3s serve as scaffolds to bring two different regions of the same protein into proximity within a single complex or two different proteins together [29,30]. By coincidence, CCA1-like proteins (such as AtCCA1 and AtLHY, GmMYB176 and GmMYB62) are often required to form hetero- and homodimers to presumably recognize DNA with high affinity and specificity, therefore regulating their downstream genes [29,31]. Recently, we revealed that soybean 14-3-3s affect isoflavonoid biosynthesis via regulating intracellular localization of a CCA1-like transcription factor, GmMYB176 [23]. In addition, 14-3-3 proteins serve as scaffolds for GmMYB176 and another CCA1-like protein (GmMYB62), and modulate their intracellular localization in soybean [29]. These findings indicated that 14-3-3s might be important players in regulating activities of CCA1-like transcription factors. Thus, exploring the interaction between CCA1-like proteins and 14-3-3s is an advisable way to address the regulation of CCA1-like MYB activities.

The whole genome sequencing has greatly contributed to the survey of plant CCA1-like proteins. For example, data mining has identified 20 and 23 CCA1-like genes in Arabidopsis and rice genome, respectively [3]. Soybean (*Glycine max*) is an important grain legume for seed protein and edible vegetable oil, and a predominant source of isoflavonoids and saponins. In total, 342 MYB-related genes from *Glycine max* have been released in PlantTFDB 4.0 (Available online: http://planttfdb.cbi.pku.edu.cn/), which provides much valuable information for understanding soybean CCA1-like proteins. To date, only few CCA1-like MYBs have been well-characterized and studied functionally in soybean including 4 CCA1-like genes (*Glyma*.*03G261800*, *Glyma*.*07G048500*, *Glyma*.*16G017400*, and *Glyma*.*19G260900*) involved in the regulation of flowering and/or seed development in a circadian clock-independent manner [32,33]; two CCA1-like genes (*GmMYB176* and *GmMYB62*) functioning together for gene regulation in soybean [29]; *GmMYB177* conferring stress tolerance in transgenic Arabidopsis [34]. In this study, we conducted a genome-wide identification of CCA1-like proteins in soybean, and presented their evolutionary characterizations and transcript profiles. Further, one of soybean CCA1-like proteins, GmMYB138a, was functionally characterized. Protein–protein interaction assay indicated that GmMYB138a interacts with a 14-3-3 protein (GmSGF14l), which leads to alteration of the intracellular localization of GmMYB138a. Additionally, qPCR analysis suggested that *GmMYB138a* and *GmSGF14l* synergistically or antagonistically respond to abiotic stresses.

## 2. Results

### 2.1. Identification of Soybean CCA1-Like Proteins and Their Phylogenetic Relationships

To identify CCA1-like proteins in soybean, the sequences of 342 MYB-related proteins (1R-type MYB) from *Glycine max* were downloaded from PlantTFDB 4.0 (http://planttfdb.cbi.pku.edu.cn/). Meanwhile, soybean putative MYB proteins were identified using a HMM search (PF00249) and a key word search against the soybean databases, and 1R-type MYBs were subsequently isolated from the putative MYB sequences using SMART analysis. The consensus sequence SHAQK(Y/F)F was used as query to search CCA1-like proteins against all 1R-type MYB sequences. These processes identified 54 CCA1-like proteins. The detailed information regarding all the 54 CCA1-like proteins is listed in Table S1. Briefly, the deduced proteins contain 91 to 1123 amino acid residues (aa) with the calculated molecular weights from 10.40 to 127.16 kDa, and their isoelectric points were estimated from 5.55 to 10.28. All the CCA1-like proteins harbor one MYB domain, and the consensus sequence SHAQK(Y/F)F is localized within the MYB repeat in each protein (Table S1). Besides, 12 CCA1-like proteins have one ZnF_C2HC motifs at N terminal, which is recognized to bind DNA, RNA, protein and/or lipid substrates [35,36]. Additionally, their subcellular localizations were predicted using pSORT software. It was found that, although CCA1-like proteins might localize in nucleus, they still have potential to be targeted to other organelles such as cytoplasm, peroxisome, chloroplast and/or mitochondria (Table S1).

**Figure 1:**
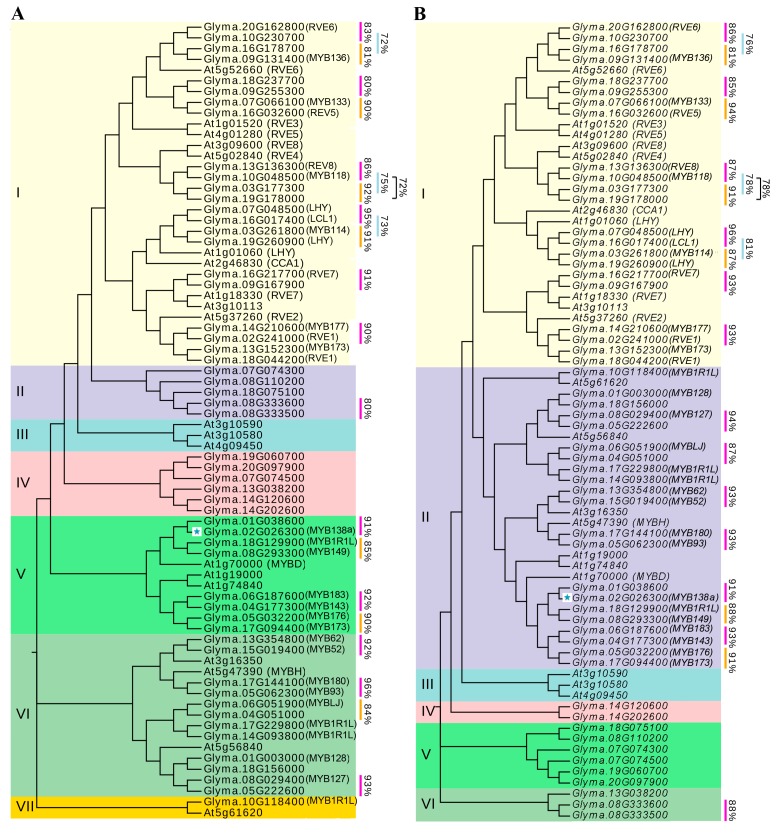
Phylogenetic analysis of CCA1-like subfamily between soybean and Arabidopsis. Phylogenetic trees were generated using: protein matrix (**A**); and CDS (Coding sequence) matrix (**B**), and groups are indicated by roman letters (I-VII/VI). The percent identity between duplicated gene pair is listed, and GmMYB138a (Glyma.02g026300) is marked with a star.

To examine the evolutionary relationship of CCA1-like subfamily between soybean and other species, a phylogenetic analysis was performed at both protein and nucleotide levels. As shown in Figure 1, trees with similar topologies were generated in both the cases, and these proteins and their corresponding genes were grouped into 6 and 7 classes, respectively. Both phylogenetic trees showed that majority of soybean CCA1-like subfamily members can be clustered together with one or more Arabidopsis CCA1-like protein(s). For example, 22 soybean CCA1-like proteins were grouped together with CCA1, LHY and RVEs in the class I, while Glyma.17g144100 and Glyma.05g062300 formed a branch together with MYBD, suggesting a conservation property of CCA1-like subfamily among different plant species. However, six soybean CCA1-like proteins (such as Glyma.07g074500, Glyma.13g03800, Glyma.14g120600, Glyma.14g202600, Glyma.19g060700, and Glyma.20g097900) and three Arabidopsis CCA1-like proteins (such as At3g10590, At3g10580, and At4g09450) formed independent clades in phylogenetic tree, respectively (the clades III and IV in Figure 1A, the clades III and V in Figure 1B), implying that specificity of CCA1-like subfamily might exist among plant species. Additionally, the analysis also grouped two soybean CCA1-like proteins with high sequence identity into the same discrete clade in the phylogenetic trees, for example Glyma.10g230700 and Glyma.20g162800 with 83% and 86% sequence identity, and Glyma.17g144100 and Glyma.05g062300 with 96% and 93% sequence identity at protein and nucleotide levels, respectively (Figure 1, Tables S2 and S3), suggesting that these gene pairs might be two closely-related orthologs in soybean.

### 2.2. Chromosomal Localizations and Duplications of CCA1-Like Genes in Soybean

**Figure 2:**
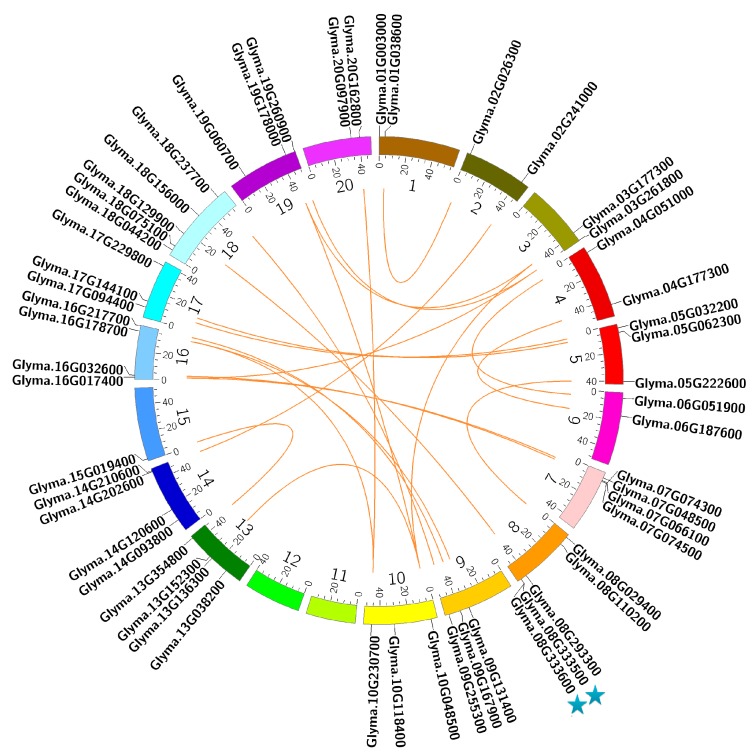
Chromosomal localization and duplication of CCA1-like genes in soybean. Each colored box refers to a chromosome, and chromosome numbers are shown beside each chromosome. The approximate localization of each soybean CCA1-like gene is indicated by a short black line. Tandem duplication is marked with star. The orange lines indicate the linkage group with segmentally duplicated gene pairs, and segmental duplication regions were estimated using the Plant Genome Duplication Database.

The chromosomal localizations of 54 CCA1-like genes were determined by their genomic distributions on soybean chromosome. It was found that these CCA1-like genes were distributed unevenly on 18 chromosomes in soybean (Figure 2 and Table S1). Some chromosomes harbor a relatively high number of CCA1-like genes while others contain very few. For example, five CCA1-like genes are distributed on the chromosomes 8 and 18, followed by four genes on the chromosomes 7, 13, 14 and 16, whereas only one CCA1-like genes is present on the chromosome 15 (Figure 2 and Table S1). None of CCA1-like genes is located on the chromosomes 11 and 12.

Tandem amplification and segmental duplication of chromosomal regions are main contributors for gene extension during evolution [37]. In general, tandem amplification refers to two paralogs dispersed by less than five genes on the same chromosome [38]. It was found that two gene pairs (Glyma.07G074300 and Glyma.07G074500, and Glyma.08G333500 and Glyma.08G333600) were separated by the genomic region of 9987 bp and 11,771 bp, respectively (Table S1). Furthermore, 88% and 80% sequence identity at nucleotide and protein levels was observed between Glyma.08G333500 and Glyma.08G333600, respectively, whereas the corresponding identities between Glyma.07G074300 and Glyma.07G074500 were very low (Tables S2 and S3). These results suggested that Glyma.08G333500 and Glyma.08G333600 have undergone tandem amplification during evolution. Based on coordinates of CCA1-like genes, we further investigated whether segmental duplications contributed to the expansion of CCA1-like subfamily in soybean. As shown in Table S4 and Figure 2, 22 duplication sets covering 38 CCA1-like genes were mapped on 19 distinct duplicate blocks. It is noteworthy that 22 duplication sets of CCA1-like genes were clustered into a discrete clade in phylogenetic tree with 76–96% and 72–96% identity at nucleotide and protein level (Figure 1), respectively, suggesting that these pairs of CCA1-like genes on the same block are possibly originated from segmental duplication events during evolution. To understand the selective evolutionary pressure on the divergence of CCA1-like genes after duplication, the non-synonymous/synonymous substitution ratio (Ka/Ks) was extracted for the 22 duplicated gene pairs from Plant Genome Duplication Database. Consequently, their Ka/Ks values varied from 0.14 to 0.57 (Table S4). The low Ka/Ks values suggested that these duplicated gene pairs might have undergone a purifying selection with limited functional divergence after duplication.

### 2.3. Tissue-Specific Expression Patterns of CCA1-Like Genes in Soybean

**Figure 3:**
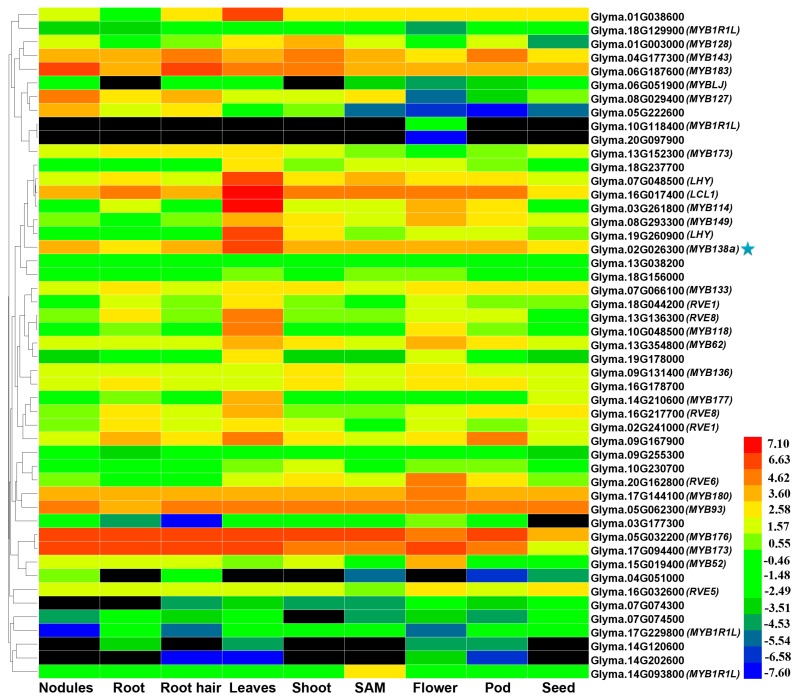
Expression patterns of CCA1-like genes in soybean. The transcript profiling data of CCA1-like genes in different soybean tissues were retrieved from Phytozome database (Available online: http://www.phytozome.net) for heat map generation. The colored scale bar beside the heat map indicates gene expression level. SAM, shoot apical meristem. *GmMYB138a* (Glyma.02g026300) is marked with a star.

To investigate the expression patterns of the 54 CCA1-like genes, we retrieved the publicly available transcript profiling data of soybean tissues from the Phytozome database (http://www.phytozome.net), including RNAseq reads from six vegetative tissues (nodule, root, root hair, stem, leaf, and shoot apical meristem) and three reproductive tissues (flower, pod, and seed). As shown in Figure 3, soybean CCA1-like genes displayed tissue-specific expression patterns. To assess the expression patterns, the maximal fold change for each of CCA1-like genes was calculated using the ratio of the maximal and minimal FPKM in different tissues. Consequently, the maximal fold changes varied from 1.8 to 1908.2, and 30 CCA1-like genes showed the maximal fold change with the value of more than five folds (Table S5). In addition, it was found that 17 CCA1-like genes had the highest accumulation of transcripts in leaf, 13 genes in flower, three genes in stem, 10 genes in root, four genes in nodule, and one gene in shoot apical meristem and seeds (Figure 3 and Table S5). It is noteworthy that 10 CCA1-like genes were highly expressed in one or few tissues (*Glyma*.*01G038600*, *Glyma*.*01G003000*, *Glyma*.*03G261800*, *Glyma*.*05G222600*, *Glyma*.*08G029400*, *Glyma*.*10G048500*, *Glyma*.*16G017400*, *Glyma*.*19G178000*, *Glyma*.*19G260900*, and *Glyma*.*20G162800*), suggesting their potential functions in the specific tissue(s) (Figure 3). For instance, Glyma.03G261800 was mainly expressed in leaves, whereas its transcript accumulation was very low in the other eight tissues (Figure 3 and Table S5), suggesting its roles in leaf-related processes. Similarly, Glyma.08G029400 exhibited distinct expression in nodules as compared to the ones in other tissues, implying that this gene might be involved in the regulation of nodule-related processes.

Five CCA1-like genes (*Glyma*.*08G333600*, *Glyma*.*08G110200*, *Glyma*.*08G333500*, *Glyma*.*18G075100* and *Glyma*.*19G060700*) were undetectable in all the nine tissues (Table S5). Additionally, a blast search was performed against soybean Expressed Sequence Tags (ESTs) in NCBI database using their CDS sequences as queries. However, no soybean ESTs were found to correspond to these five genes, suggesting that they might be pseudogenes or transcribed at specific developmental processes or under special conditions.

### 2.4. GmMYB138a Interacts with GmSGF14l

**Figure 4:**
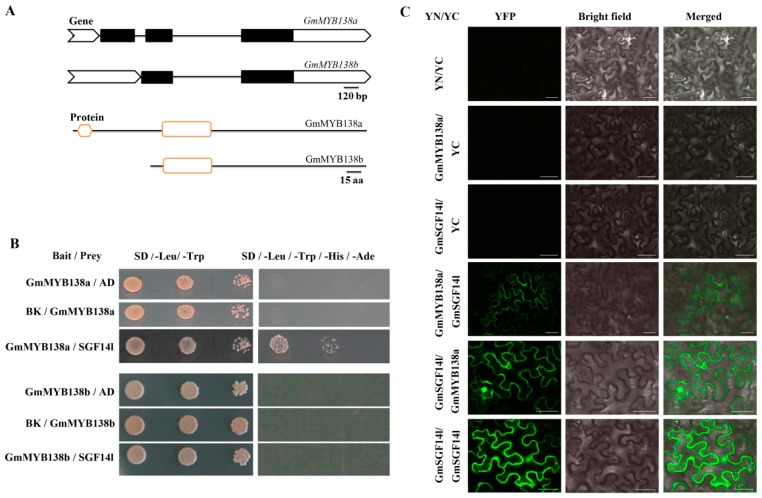
Interaction of GmMYB138a with GmSGF14l. (**A**) Schematic diagram for two spliced transcripts of Glyma.02g026300 (GmMYB138a and GmMYB138b) at protein and gene levels. Black lines represent the introns, while black boxes refer to the exons of two spliced transcripts in the gene structures. In the protein structures, amino acids are indicated by black line. MYB domain is marked with rectangle, while sexangle refer to ZnF_C2HC domain; (**B**) The investigation of interaction between GmSGF14l and GmMYB138a/b using yeast two-hybrid assay. Yeast cells were co-transformed with combination of DNA-binding domain (BK, Bait) and activation domain (AD, Prey) fused constructs as indicated. A series of 5 µL of diluted yeast suspension culture co-transformed with bait and prey constructs was spotted onto synthetic defined (SD) selection plates. Growth on SD/-Leu/-Trp indicates the presence of both the vectors, while growth on SD/-Leu/-Trp/-His/-Ade shows interaction between prey and bait. The different combinations with GmMYB138a or GmMYB138b/BK or AD are negative controls; (**C**) The BiFC assay showing interaction between GmYB138a and GmSGF14l. Tobacco leaves were co-transformed with GmYB138a and GmSGF14l proteins fused to the N- or C-terminal half of yellow fluorescent protein followed by confocal microscopy. Scale bar is shown in 40 μm.

Previously, we demonstrated that GmMYB176 (Glyma.05G032200) and GmMYB62 (Glyma.13G354800) can interact with a 14-3-3 protein (GmSGF14l) in soybean [29]. Both GmMYB176 and GmMYB62 are CCA1-like proteins with ZnF_C2HC domain. To identify additional CCA1-like proteins that interact with GmSGF14s, additional CCA1-like proteins with ZnF_C2HC domain were subjected to a targeted Y2H assay where GmSGF14l was used as the representative of 14-3-3 family. Consequently, GmSGF14l can directly interact with GmMYB138a (Glyma.02G026300.1), and its corresponding structures at gene and protein levels were indicated in Figure 4A. As shown in Figure 4B, yeast colony growth was observed on SD/-Leu/-Trp/-Ade/-His when the plasmids containing GmMYB138a as bait and GmSGF14l as prey were co-transformed, while yeast colony in negative controls did not grow on SD/-Leu/-Trp/-Ade/-His. This result indicated that physical interaction occurred between GmMYB138a and GmSGF14l. Intriguingly, the locus of Glyma.02G026300 has the ability of encoding two MYB proteins (GmMYB138a and GmMYB138b), and both protein sequences are the same except that the first 91 amino acids of GmMYB138a were replaced by 13 amino acids in GmMYB138b (Figure 4A). As compared with GmMYB138a, GmMYB138b is shorter and lacks ZnF_C2HC domain.

We also cloned the gene *GmMYB138b* and examined its interaction with GmSGF14l. Unexpectedly, no yeast colony can grow on SD/-Leu/-Trp/-Ade/-His (Figure 4B), indicating that GmMYB138b did not interact with GmSGF14l. Furthermore, binding sites of 14-3-3 in GmMYB138a/b were predicted using the MotifScan tool (Available online: http://scansite.mit.edu/motifscan_seq.phtml). pST-binding motif is a potential binding site for a 14-3-3 protein, and Ser or Thr residues in the motifs are potentially phosphorylated [10]. It was found that GmMYB138a harbors six potential pST-binding motifs (T19 (GHNSRTCtDGGAAAS), T45 (LFGVRVMtEANSSFR), S56 (SSFRKSAsMNNLSQY), S119 (KGDWRGIsRNFVKTR), and S152/153 (QNRRRRRssLFDITT)), whereas GmMYB138b only contains three pST-binding motifs (S41 and S74/75), which correspond to S119 and S152/153 in GmMYB138a, respectively (Figure S1). These results suggested that the pST-binding motifs (T19, T45 and T56) within GmMYB138a might be very important for binding of 14-3-3 proteins.

To confirm the interaction between GmMYB138a and GmSGF14l in planta, a BiFC analysis was conducted as described in detail previously [39]. Translational fusion of *GmMYB138a* or *GmSGF14l* was generated in the binary vector that contained either N-terminus half (YN) or C-terminus half (YN) of YFP. The negative controls included the following combinations: (i) two non-fusion halves of YFP, YN and YC; (ii) GmMYB138a-YN; and (iii) GmSGF14l-YN with the non-fusion YC. As shown in Figure 4C, the YFP signals were almost evenly distributed in both nucleus and cytoplasm whereas the negative controls showed no signal. This result validated that GmSGF14l can interact with GmMYB138a in planta.

### 2.5. Functional Characterization of GmMYB138a

**Figure 5:**
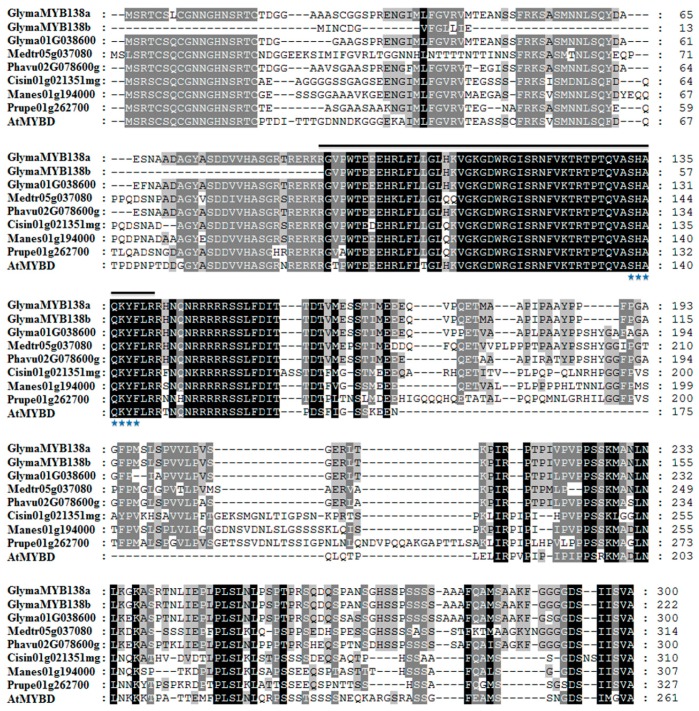
Multiple sequence alignment of deduced amino acid sequence of GmMYB138a with its homologs in different species. Amino acid sequences were aligned by ClustalW, and imported in Genedoc for shading. Identical amino acid residues are shown in black. If the conserved percent was up to 80% and 60%, the amino acid residues were shaded with gray and light gray colors, respectively. The MYB domains of CCA1-like proteins are indicated by lines. The consensus sequence SHAQK(Y/F)F is marked with stars.

The full-length *GmMYB138a* cDNA sequence (903 nucleotides) encodes a 300 aa protein with an estimated molecular mass of 32.17 kDa, which belongs to 1R-type MYB with ZnF_C2HC domain. It is located on chromosome 2 in soybean (Figure 2) and comprises three exons and two introns (Figure 4B). The phylogenetic analysis grouped *GmMYB138a* (Glyma.02G026300) together with *GmMYB158* (Glyma.01G038600) that resides on chromosome 1. Both of them were mapped on the duplication block 8 with more than 91% sequence identity at nucleotide and amino acid levels, suggesting that they are duplicated gene pair. To obtain some hints about the functions of GmMYB138a, its most closely-related homologs were obtained from seven different plant species. Multiple sequence alignment indicated that they are highly conserved with 51.3–91.0% sequence identity (Figure 5), and the sequence diversification mainly occurs at the C-terminal regions. Noticeably, GmMYB138a shares 51.3% protein identity with AtMYBD, which was found to regulate anthocyanin accumulation in response to light and cytokinin in Arabidopsis.

**Figure 6:**
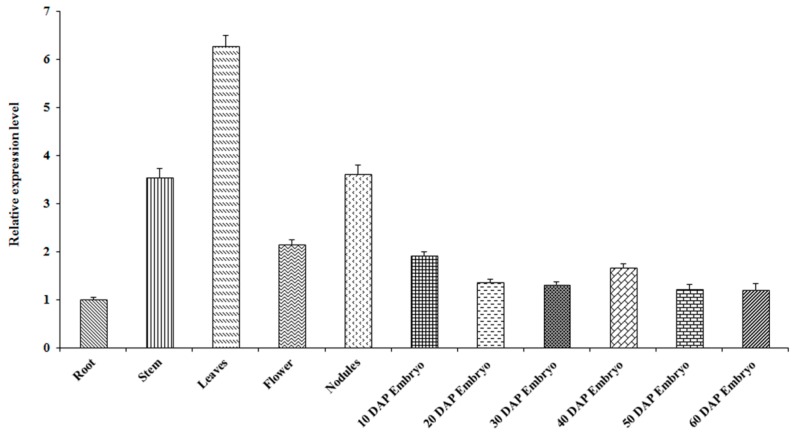
Expression analysis of *GmMYB138a* in different soybean tissues and developmental seeds. Total RNA extracted from soybean root, stem, leaf, nodule, flower, embryo (10, 20, 30, 40, 50 and 60 days after pollination) were used for qPCR analysis. Three biological replicates and three technical replicates for each biological replicate were carried out. The standard error of the mean is represented by an error bar. The data were normalized against *SUBI-3*.

To provide clues for the roles of *GmMYB138a*, its expression pattern in vegetative and reproductive tissues was investigated using qPCR approach. As shown in Figure 6, *GmMYB138a* showed the highest expression in leaves, followed by nodules and stem, and their fold changes reached 6.26, 3.61 and 3.54, respectively, as compared to the ones in root. In comparison, less expression level of *GmMYB138a* was observed in root and reproductive tissues. To explore its functional roles in seed development, the seeds at six developmental stages (10, 20, 30, 40, 50, and 60 days after pollination) were also used to examine the expression of *GmMYB138a*. As shown in Figure 6, *GmMYB138a* remains a low and stable expression level during seed development, suggesting that it might not be a main player in the regulation of seed development. Furthermore, promoter analysis indicated that several cis-acting regulatory elements responsive for auxin, gibberellin and salicylic acid exist in the promoter region of *GmMYB138a*. Interestingly, two circadian cis-acting regulatory elements involved in circadian control were observed, supporting its CCA1-like property [6]. Additionally, its promoter region contains TC-rich repeats, a cis-acting element involved in defense and stress responsiveness.

**Figure 7:**
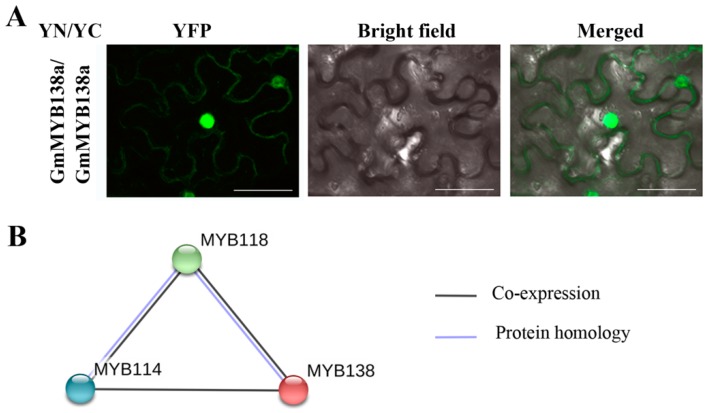
Dimer formation of GmMYB138a in planta: (**A**) BiFC analysis showing homodimer formation of GmMYB138a in vivo; and (**B**) prediction of GmMYB138a heterodimer using STRING. MYB114, Glyma.03g261800; MYB118, Glyma.10g048500. Scale bar is shown in 40 μm.

1R-type MYBs form hetero- and homodimers to presumably recognize DNA with high affinity and specificity [2,40]. To investigate if GmMYB138a can form homodimer in planta, BiFC analysis was performed as described above. Consequently, the YFP signals were clearly observed in nucleus and cytoplasm, suggesting that GmMYB138a can interact with itself in planta (Figure 7A). YFP signals for the homodimers of GmMYB138a were mainly distributed in nucleus, indicating that GmMYB138a was predominantly localized in nucleus. To explore if GmMYB138a can form heterodimers with other GmMYBs, an interaction prediction was conducted against the protein database of *Glycine max* using STRING (https://string-db.org/). As shown in Figure 7B, GmMYB138a was predicted to interact with GmMYB114/GmLCL2 (Glyma.03g261800) and GmMYB118 (Glyma.10g048500), and both proteins belongs to CCA1-like subfamily.

### 2.6. Expression Patterns of GmMYB138a and GmGF14l in Response to Abiotic Stresses

Several pieces of evidence indicated that CCA1-like genes might be involved in plant stress responses [11,16,21]. To explore if *GmMYB138a* performs function in response to stresses, we examined its expression in whole seedlings under the stresses of cold, drought and salinity.

**Figure 8:**
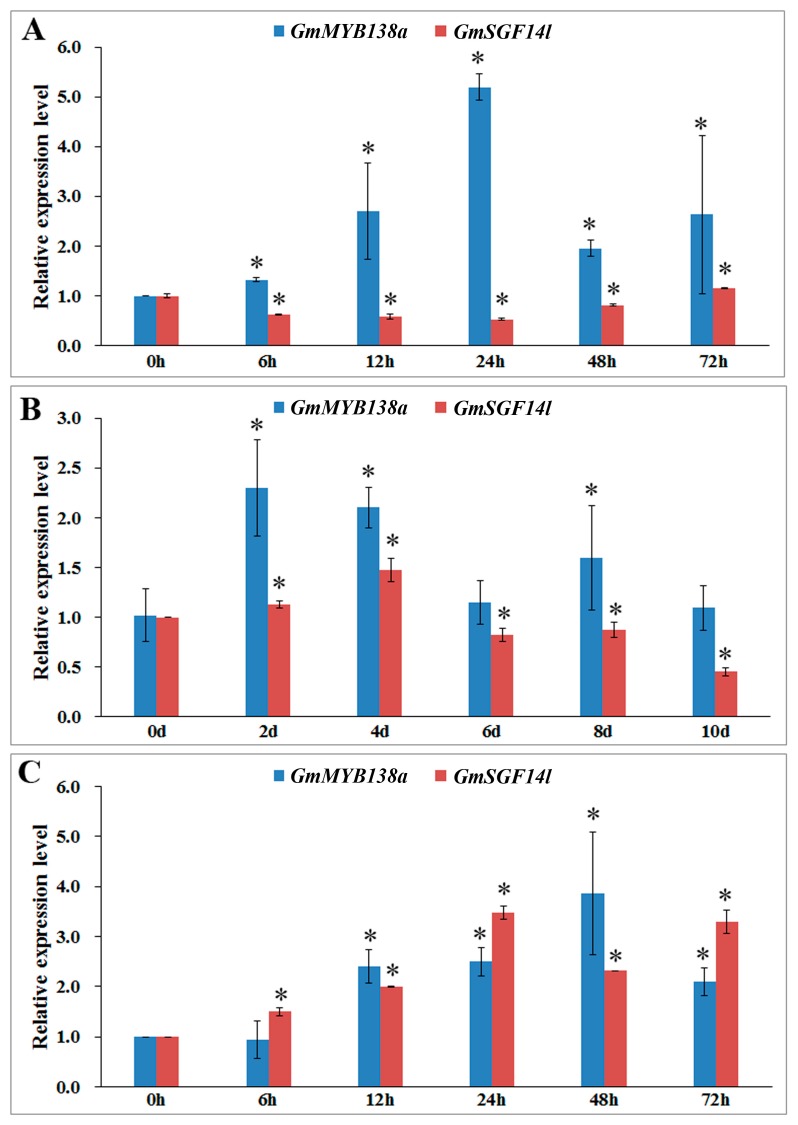
Expression analysis of *GmMYB138a* and *GmSGF14l* in response to abiotic stresses. Ten-day-old soybean seedlings were exposed to stress treatment as indicated below. Gene expression analysis was conducted by qRT-PCR using gene specific primers. (**A**) Gene expression pattern of *GmMYB138a* and *GmSGF14l* in seedlings exposed to cold stress for 0, 6, 12, 24, 48 and 72 h; (**B**) Gene expression pattern of *GmMYB138a* and *GmSGF14l* in seedlings exposed to drought stress for 0, 2, 4, 6, 8 and 10 days; (**C**) Gene expression pattern of *GmMYB138a* and *GmSGF14l* in seedlings exposed to salinity stress for 0, 6, 12, 24, 48 and 72 h. Error bars indicate SE (Standard error) of two biological and three technical replicates. Values were normalized against *SUBI-3*. Significant differences are denoted by asterisks (*p* < 0.01).

As shown in Figure 8, the expression of *GmMYB138a* was significantly increased by cold, drought and salt stresses. Briefly, the transcripts of *GmMYB138a* were accumulated to its maximum level at 24 h after cold stress with 5.2 folds increase as compared to control (Figure 8A), while a dramatic increase in *GmMYB138a* transcripts (2.3 and 2.1 folds, respectively as compared to control) was observed on Days 2 and 4 of drought treatment (Figure 8B). When young seedlings were subjected to salt stress, the expression of *GmMYB138a* was remarkably pronounced by 2.4 folds at 12 h, and then remained a relatively stable level as stress prolongs (Figure 8C).

It is well known that 14-3-3s play important roles in response to stresses in plants. Since GmSGF14l exhibited interaction with GmMYB138a, we also investigated the expression of *GmSGF14l* under the above stresses. The expression patterns of *GmSGF14l* were similar to the ones of *GmMYB138a* when subjected to drought and salinity stresses. As shown in Figure 8B,C, the expression of *GmSGF14l* was increased to its maximum level at some stress point. In contrast, *GmSGF14l* was clearly decreased from 6 h to 48 h after cold stress (Figure 8A), basically opposite to the expression pattern of *GmMYB138a* under cold stress.

## 3. Discussion

CCA1-like proteins are a group of 1R-type MYBs generally featured by a highly conserved motif SHAQK(Y/F)F in plants. They play important roles in multiple biological processes such as control of circadian rhythm, biosynthesis of isoflavonoid and anthocyanin, flowering, leaf senescence, hypocotyl elongation, seed germination, hormone signaling pathway, and stress responses [5,6,7,8,9,10,11,12,13,14,15,16,17,18,19,21]. However, the comprehensive and detailed understandings regarding CCA1-like proteins are very limited in soybean, which is an important grain legume for seed protein and edible vegetable oil. In this study, we genome-widely identified and characterized 54 CCA1-like proteins in soybean, and further took GmMYB138a as example to study the functional roles of soybean CCA1-like proteins, which laid a great foundation for further studying their regulatory roles in diverse biological processes.

It has been generally accepted that tandem amplification and segmental duplication of chromosomal regions are main contributors for gene extension during evolution [37]. In the study, 23 duplicated gene pairs covering 40 CCA1-like MYB genes were identified, including one pair derived from tandem amplification and 22 pairs from segmental duplication. Each duplicated gene pair not only formed a discrete clade in the phylogenetic tree (such as Glyma.17g144100 and Glyma.05g062300; Glyma.07g048500 and Glyma.16g017400), but also exhibited low Ka/Ks ratios (0.14–0.57) and high sequence identity (72–96%) at both nucleotide and protein level (Figure 1, Tables S2–S4), suggesting that each pair of duplicated genes possibly have the closest evolutionary relationship and share similar functions in soybean. It is not surprising since soybean had undergone whole-genome duplication event ~56.5 and 19.2 million years ago, which led to duplication of at least 75% of genes in soybean genome [41,42]. Thus far, our understandings regarding functions of CCA1-like subfamily mainly depend on the relevant researches launched in Arabidopsis, and more than half of Arabidopsis CCA1-like genes have been functionally studied such as *CCA1*, *LHY*, *MYBD*, *MYBH* as well as several *RVE*s [6,7,11,12,13,14,15,17,18,19,31]. In the study, phylogenetic analysis indicated that majority of soybean CCA1-like proteins were clustered together with their corresponding homolog(s) in Arabidopsis (Figure 1). For example, four soybean CCA1-like proteins were grouped together with AtCCA1 and AtLHY functioning as central components in circadian oscillators to control several biological processes and stress responses [6,7,11,13,16,31]. Meanwhile, two soybean CCA1-like proteins (Glyma.17g144100 and Glyma.05g062300) formed the same discrete clade with AtMYBH (Figure 1). It has been demonstrated that AtMYBH serves as a circadian oscillator to specifically modulate cell expansion during leaf development, and participates in the regulation of leaf senescence and hypocotyl elongation [20,43,44]. Similarly, 18 soybean CCA1-like proteins were clustered together with AtRVEs (Figure 1), which were found to control growth of juvenile and adult plants, seed dormancy and germination, and anthocyanin biosynthesis [15,17,19,45]. Although majority of soybean CCA1-like proteins have not been functionally annotated or characterized yet, their corresponding Arabidopsis ortholog(s) identified in this study provides clues for their possible functions. It has been proposed that gene expression provides functional specificity in certain tissues. The soybean CCA1-like genes displayed tissue-specific expression patterns. Especially, 10 CCA1-like genes were highly expressed only in one or few tissues (Figure 3 and Table S5). Diverse expression patterns in soybean tissues indicated functional diversification among CCA1-like genes. Thus, based on phylogenetic analysis and their tissue-specific expression patterns, we speculated that CCA1-like subfamily might exert diverse functions in soybean.

It has been well accepted that protein-protein interactions can affect the regulatory activity of MYB transcription factors. 14-3-3s are usually able to modulate activities of diverse target proteins via alteration of the stability, activity, intracellular localization or interaction capability of their client proteins [26,27]. Our previous studies revealed that soybean 14-3-3 proteins can interact with 2 CCA1-like transcription factors, GmMYB176 and GmMYB62, therefore regulating their downstream genes in soybean [23,29]. In the present study, we demonstrated that GmMYB138a can interact with GmSGF14l, an isoform of 14-3-3 proteins in soybean (Figure 4). Noticeably, YFP signals derived from the interaction of GmMYB138a with GmSGF14l were almost evenly distributed in both nucleus and cytoplasm (Figure 4C), whereas GmMYB138a was mainly distributed in nucleus (Figure 7A), consistent with its functional localization as transcription factor. These results are in agreement with our previous report that 14-3-3 proteins regulate the intracellular localization of GmMYB176 thereby affecting isoflavonoid biosynthesis in soybean [23]. Thus, we assumed that GmSGF14l might affect the functions of GmMYB138a via regulating its intracellular localization in soybean. Intriguingly, the locus of *GmMYB138a* has ability of generating alternatively spliced transcripts (*GmMYB138a* and *GmMYB138b*) (Figure 4A). Several pieces of evidence indicated that CCA1-like genes have divergent alternative splicing, which might exert different and often antagonistic functions [46,47,48]. For example, the locus of *CCA1* can generate alternative splices (CCA1α and CCA1β), and the binding of CCA1β to CCA1α or LHY can prevent formation of normal CCA1α homodimers, LHY homodimers, and CCA1α-LHY heterodimers, thereby reducing DNA binding affinity [46]. Our yeast two-hybrid assay revealed that GmSGF14l interacts with GmMYB138a, whereas no interaction was observed between GmSGF14l and GmMYB138b (Figure 4B). These protein–protein interaction studies supported the possibility that alternative splices of CCA1-like genes might be involved in different biological processes via interacting with different protein clients like 14-3-3s. Undoubtedly, addressing the functional roles of the two alternative splices is more interesting and significant in future.

Although plant CCA1-like proteins might perform important functions in multiple biological processes, the functional roles of *GmMYB138a* remain totally unknown in soybean. Recently, several studies indicated that CCA1-like proteins might be involved in response to various stresses [7,11,16,21,46]. Our study revealed that GmMYB138a interacts with a 14-3-3 protein, GmSGF14l. It has been well known that 14-3-3s are important players in the regulation of cellular responses to environmental stresses in plant [23,26,27,30]. In the present study, the expression patterns indicated that *GmMYB138a* and *GmSGF14l* might respond to cold, drought and salt stresses synergistically or antagonistically. Consistent with previous reports that *CCA1-like* gene expression is affected by cold, heat, osmotic, salt and oxidative stresses [49,50], the expression of *GmMYB138a* was significantly increased by cold, drought and salt stresses (5.20, 2.30 and 3.86 folds changes, respectively) (Figure 8), implying that *GmMYB138a* possibly functions as a modulator of stress-induced signaling pathways. Likewise, the expression patterns of *GmSGF14l* were also clearly increased when subjected to drought and salinity stresses (Figure 8B,C), which are in agreement with previous reports that *GsGF14o* expression was greatly induced by drought stress [51], while *BdGF14d* confers salt tolerance in transgenic tobacco plants [52]. The similarity in response to drought and salt stress supported that signaling pathway activated by drought and salt are largely overlapping in plants [53]. In addition, similar expression patterns and protein interaction suggested that *GmMYB138a* and *GmSGF14l* might be in the same pathway in response to drought and salt stresses. Unlike its response to drought and salt stresses, however, *GmSGF14l* was clearly decreased from 6 h to 48 h after cold stress (Figure 8A), consistent with previous reports that 14-3-3s can function as negative regulators of freezing tolerance and/or cold acclimation in Arabidopsis [54,55]. Although GmSGF14l and GmMYB138a can interact with each other, they were conversely expressed in response to cold stress (Figure 8A), which is consistent with or previous study expressions of two 14-3-3 genes (*PvGF14e* and *PvGF14h*) were increased by cold stress and decreased by salinity and drought stress [56]. We assume that they are possibly involved in different pathways responsive for cold tolerance.

## 4. Materials and Methods

### 4.1. Plant Materials

Soybean (*Glycine max* cv. Jilin 32) plants were grown at an experimental station in Jilin University (Changchun, Jilin, China), in 2016. Soybean tissues were collected from 5–10 random plants including nodules, root, leaves, stem, flower, and seeds at six developmental stages (10, 20, 30, 40, 50, 60 days after flowering), frozen in liquid nitrogen and stored at −80 °C. *Nicotiana benthamiana* plants were grown in pots under 16 h light at 25 °C and 8 h dark at 20 °C with 70–80% relative humidity.

Abiotic stresses were conducted as described in detail previously [57]. Briefly, ten-day-old seedlings (soybean cultivar “Jilin32”) were subjected to the following treatments and six pots of seedlings were used for each treatment: (1) for cold stress, seedlings were transferred to 4 °C and samples were collected at 0, 6, 12, 24, 48 and 72 h after cold treatment; (2) for drought stress, water supply was withheld and samples were collected at 0, 2, 4, 6, 8 and 10 days of water stress; and (3) for salinity stress, 200 mM NaCl solution was applied to seedlings and samples were collected at 0, 6, 12, 24, 48 and 72 h after salt treatment. The aboveground parts were collected and frozen in liquid nitrogen, and stored at −80 °C.

### 4.2. In silico Analysis

To identify CCA1-like proteins in soybean, the sequences of MYB-related proteins (1R-type MYB) in *Glycine max* were downloaded from PlantTFDB 4.0 (Available online: http://planttfdb.cbi.pku.edu.cn/). Meanwhile, a keyword search using the word “MYB” was also conducted against the phytozome 12 *Glycine max* Wm82.a2.v1 database (Available online: www.phytozome.net), and 843 putative MYB proteins were obtained. To fish out more MYB proteins, the MYB profile (PF00249) was used to conduct HMM search against *Glycine max* database (Available online: http://www.ebi.ac.uk/Tools/hmmer/search/hmmsearch), and the parameters were set as default. Consequently, 803 putative MYB proteins were found. The putative MYB proteins obtained from HMM search and keyword search were combined, and the redundant sequences were removed. Subsequently, 1R-type MYBs were isolated from the above non-redundant MYB sequences using SMART analysis (Available online: http://smart.embl-heidelberg.de/). Following the method used by Du et al. [5], the consensus sequence SHAQK(Y/F)F was used as query to search CCA1-like proteins against all the 1R-type MYB sequences. Additional domains other than MYB were identified using the SMART database. Their subcellular localizations were predicted using pSORT algorithms with default parameters (Available online: http://www.psort.org/).

### 4.3. Multiple Sequence Alignment and Phylogenetic Analysis

The sequences of CCA1-like proteins and genes in Arabidopsis were downloaded from TAIR (Available online: http://www.arabidopsis.org/). All the CCA1-like protein sequences as well as gene sequences from soybean and Arabidopsis were aligned using ClustalW, respectively, and the alignment was imported to MEGA7 for phylogenetic analysis. The phylogenetic trees were generated using neighbor-joining method with the midpoint and the p-distance model [58]. The bootstrap value was assessed with 1000 replicates. The phylogenetic trees were visualized by the online Evolview program (Available online: http://www.evolgenius.info/evolview/).

### 4.4. Chromosomal Localization and Gene Duplication

The gene location and chromosome number for each of soybean CCA1-like genes was retrieved from phytozome 12 *Glycine max* Wm82.a2.v1 database (Available online: www.phytozome.net). The chromosomal location image of soybean CCA1-like genes was generated by the Circos software. As described in detail previously [39], CCA1-like genes in duplicated genomic regions and Ka/Ks values for each duplicated genes were obtained for syntenic mapping from batch download option of Plant Genome Duplication Database (Available online: http://chibba.agtec.uga.edu/duplication/). Generally, the homologous genes on the same duplicated chromosomal blocks were set as segmental duplication, while two paralogs with less than 5 gene loci in-between were defined as tandem duplication. Ka/Ks < 1 refers to purifying selection during evolution, while Ka/Ks > 1 indicates positive selection to accelerate evolution [38].

### 4.5. Gene Expression Analysis

To evaluate the expressions of soybean CCA1-like genes in different tissues, the fragments per kilobase of transcript per million mapped reads (FPKM) values for each CCA1-like gene were downloaded from Phytozome database (Available online: http://www.phytozome.net) by tracking nine tissue gene-level expression, and the data were normalized across tissues. The heat map was generated using the software HemI1.0 [59].

Total RNA was isolated from soybean tissues using RNAprep Pure Plant Kit (Tiangen, China). First strand cDNA was synthesized using PrimeScript RT reagent kit with gDNA Eraser (Takara, Kusatsu, Japan). qPCR was subsequently conducted with an ABI StepOnePlus PCR system and SYBR Premix Ex Taq (Takara). Soybean *UBIQUITIN-3* (*SUBI3*) was set as an internal reference for data normalization, and data were analyzed by the software ABI StepOnePlus v2.3. Three biological replicates were performed for the analysis of tissue-specific gene expression, while two biological replicates were conducted for the three abiotic stresses. Three technical replicates were used for each biological replicate. The primer sequences are listed in Table S6. Statistical significance of the data was analyzed by one-way ANOVA with LSD test, and *p* value < 0.05 was considered to be statistically significant.

For promoter analysis, a 1500 bp interval upstream of the transcription start site of *GmMYB138a* was analyzed in the PlantCARE database (Available online: http://bioinformatics.psb.ugent.be/webtools/plantcare/html/).

### 4.6. Targeted Yeast Two-Hybrid (Y2H) Assay

To conduct the Y2H hybrid assay, full-length cDNA of *GmMYB138a*, *GmMYB138b* and *GmSGF14l* were PCR amplified using gene-specific primers (Table S6) and cloned into the Gateway entry vector pDONOR207 by homologous recombination. The entry clones were recombined into the Y2H destination vectors pGBKT7-DEST (bait) and pGADT7-DEST (prey) to obtain pGBKT7-*GmMYB138a*, pGBKT7-*GmMYB138b*, pGBKT7-*GmSGF14l*, pGADT7-*GmMYB138a*, pGADT7-*GmMYB138b* and pGADT7-*GmSGF14l* [60]. As described in detail previously [39], the vectors in different combinations were co-transformed into yeast strain AH109 and selected on SD/-Leu/-Trp agar plates. Subsequently, yeast co-transformants were grown in liquid medium, and 5 μL AH109 culture with a series of 10 folds dilution was dropped onto SD/-Leu/-Trp and SD/-Ade/-His/-Leu/-Trp plates and grown for 5 days at 30 °C. The different combinations with GmMYB138a or GmMYB138b/BK or AD are negative controls.

### 4.7. Bimolecular Fluorescence Complementation (BiFC) Assay

For BiFC assay, the entry clones with *GmMYB138a* and *GmSGF14l* were recombined into the BiFC vectors pEarlyGate201-YN or pEarlyGate202-YC (pEG201-YN or pEG202-YN), respectively [60]. As described in detail previously [39], *Agrobacterium tumefaciens* (GV3101) cultures containing pEG201-*GmMYB138a*-*YN* and pEG202-*GmSGF14l*-*YC* or pEG201-*GmSGF14l*-*YN* and pEG201-*GmMYB138a*-*YC* were mixed 1:1 and co-transformed into tobacco leaf epidermal cells by infiltration. Epidermal cell layers of *N. benthamiana* leaves were assayed for YFP fluorescence 2 days after infiltration using Leica confocal microscope (Available online: http://www.leica.com/).

### 4.8. Predictions of pST-Binding Motif and Protein Interaction

pST-binding motifs were predicted using the MotifScan tool (Available online: http://scansite.mit.edu/motifscan_seq.phtml), and the parameters were set as 14-3-3 Mode 1 and low stringency. Interaction prediction was performed against the database of *Glycine max* using STRING (Available online: https://string-db.org/).

## 5. Conclusions

This study presents a comprehensive characterization of soybean CCA1-like proteins including their tissue-specific expression patterns and evolutionary relationships. GmMYB138a was further functionally characterized as a representative of soybean CCA1-like proteins. Our results indicated that GmSGF14l (a 14-3-3 protein) interacts with GmMYB138a to alter its subcellular localization. In addition, *GmSGF14l* and *GmMYB138a* might respond to abiotic stresses synergistically or antagonistically. These findings laid a foundation for future research regarding functions of soybean CCA1-like proteins, especially regulatory mechanisms of *GmMYB138a* in response to abiotic stresses.

## Supplementary Materials

Supplementary materials can be found at www.mdpi.com/1422-0067/18/10/2040/s1.
