# Supplementary material for: Genome-Wide Analysis of CCA1-Like Proteins in Soybean and Functional Characterization of GmMYB138a
Source: Int J Mol Sci. 2017 Sep 22;18(10):2040. doi: 10.3390/ijms18102040 (PMC5666722; doi:10.3390/ijms18102040)
Supplement: Supplementary file 1 [file ijms-18-02040-s001.zip › supplementary.pdf]

Supplementary Materials

Figure S1: Prediction of 14-3-3 binding site in GmMYB138a and GmMYB138b

Table S1: Characterization of CCA1-like proteins in soybean

Table S2: Statistic report of CCA1-like proteins in soybean

Table S3: Statistics report of CCA1-like genes in soybean

Table S4: Segmental duplication events of soybean CCA1-like genes during evolution

Table S5: The FPKM values of CCA1-like genes in different tissues

Table S6: Primers used in the study
