# Supplementary figures and images for: Genome-Wide Analysis of CCA1-Like Proteins in Soybean and Functional Characterization of GmMYB138a
Source: Int J Mol Sci. 2017 Sep 22;18(10):2040. doi: 10.3390/ijms18102040 (PMC5666722; doi:10.3390/ijms18102040)

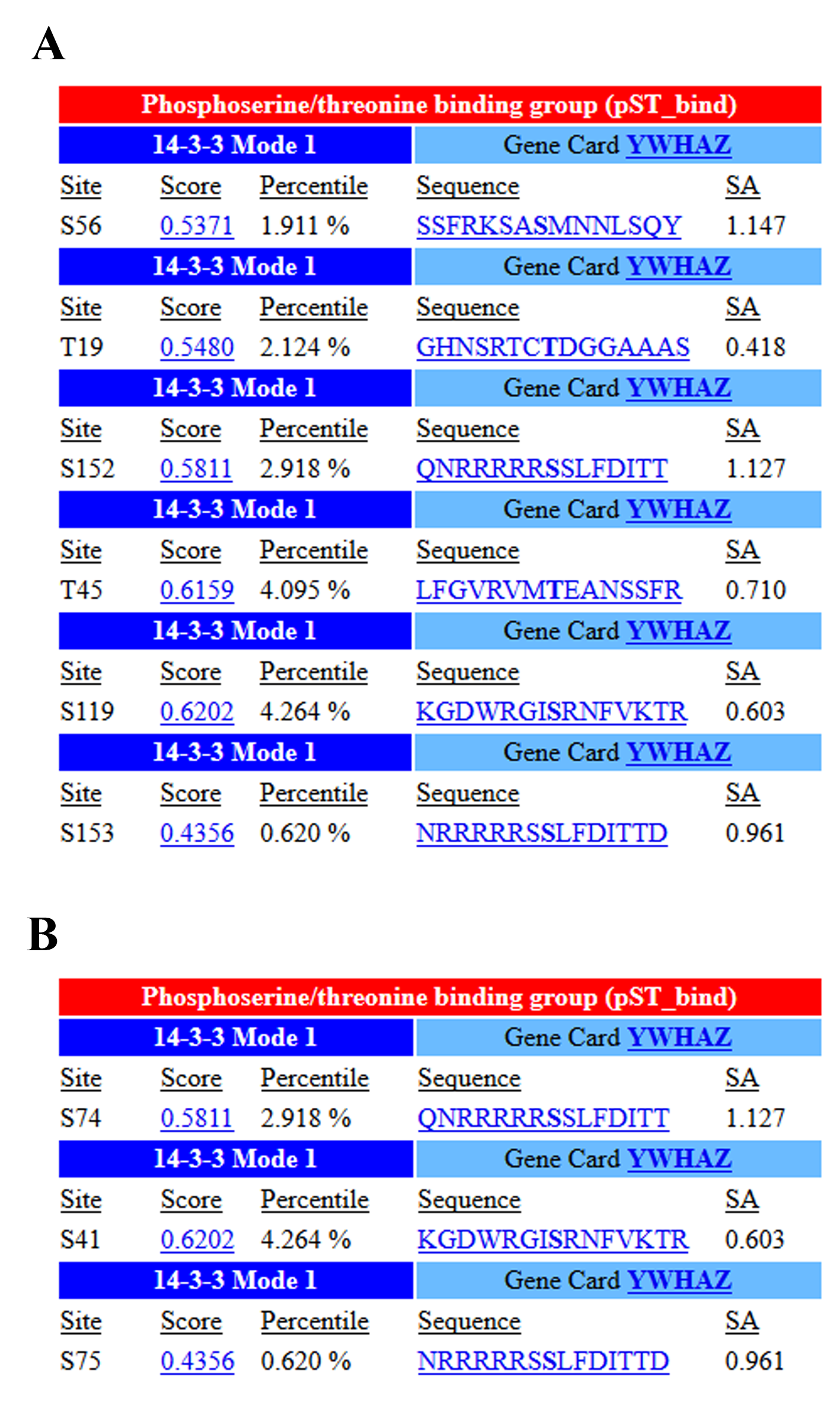

Supplement: Supplementary file 1 [file ijms-18-02040-s001.zip › Fig S1.tif]
